# Non‐ Conventional Agents Enhance Sweet Pepper (*Capsicum annuum* L. var. *annuum*) Defense against *Aphis Gossypii*, *Thrips Tabaci*, and Their Predators *Chrysoperla Carnea* and *Orius Insidiosus*

**DOI:** 10.1002/gch2.202500590

**Published:** 2026-01-31

**Authors:** Mohamed S. Zayed, Mahmoud A. Hegab, Moustafa S. SaadAllah, El‐Said M. Elnabawy, Hossam S. El‐Beltagi, Atef Abo‐Ogiala, Amged El‐Harariy

**Affiliations:** ^1^ Department of Plant Protection Faculty of Agriculture Damietta University Damietta Egypt; ^2^ Department of Pesticides Chemistry and Toxicology Faculty of Agriculture Kafrelsheikh University Kafrelsheikh Egypt; ^3^ Department of Economic Entomology Faculty of Agriculture Kafrelsheikh University Kafrelsheikh Egypt; ^4^ Agricultural Biotechnology Department College of Agriculture and Food Sciences King Faisal University Al‐Ahsa Saudi Arabia; ^5^ Horticulture Department Faculty of Agriculture Tanta University Tanta Egypt; ^6^ Department of Crop and Animal Sciences Albrecht Daniel Thaer‐Institute of Agricultural and Horticultural Sciences Faculty of Life Sciences Humboldt‐Universität Zu Berlin Berlin Germany; ^7^ Plant Protection Department Desert Research Center Cairo Egypt

**Keywords:** aphids and thrips, effective microorganisms, integrated pest management (IPM), salicylic acid, sweet pepper (Capsicum annuum)

## Abstract

A study conducted in Egypt evaluated the effectiveness of chemical and microbial agents in enhancing sweet pepper (*Capsicum annuum* var. annuum) defenses against major pests *Aphis gossypii* and *Thrips tabaci* and their natural predators, *Chrysoperla carnea* and *Orius insidiosus*. Five foliar treatments were tested under greenhouse conditions during the 2022 and 2023 growing seasons: salicylic acid (SA), potassium phosphite (PK), effective microorganisms (EMs), insecticide imidacloprid (IMI), and a water‐sprayed untreated check (control). In addition to monitoring pest and predator populations, several biochemical parameters were assessed in pepper leaves, including total chlorophyll, macronutrients (N, P, K), phenolic compounds, and total protein content. All treatments improved plant growth and enhanced physiological traits, promoting both vegetative development and fruit productivity. Salicylic acid was the most effective treatment, significantly increasing chlorophyll levels, nutrient uptake, phenolic content, and total proteins. Moreover, all agents led to a marked reduction in pest populations while preserving predator abundance, indicating strong plant defense activation and selectivity toward beneficial insects. These findings demonstrate that the tested agents, particularly salicylic acid, can serve as practical and sustainable alternatives to synthetic insecticides and are suitable for integration into environmentally friendly pest management strategies and Integrated Pest Management (IPM) programs.

## 1 Introduction

Sweet pepper (*Capsicum annuum* L.) is a widely cultivated crop globally, valued for its vibrant color, pungency, distinctive flavor, and aroma [1]. Despite its importance, sweet pepper is highly susceptible to a range of pests, including bacterial and fungal pathogens, as well as insect pests such as aphids and thrips, all of which can significantly impact yield and quality [2, 3, 4]. Sweet pepper is particularly vulnerable to diverse biotic stresses, and the extensive reliance on conventional chemical control has raised serious environmental and ecological concerns, emphasizing the need for alternative and sustainable disease and pest management strategies [5, 6]. To mitigate these threats, integrated pest management (IPM) strategies are implemented, combining microbial and chemical control methods with plant defense inducers and other complementary approaches [7].

Among the major threats to pepper crops are sap‐sucking insects such as aphids and thrips. *Aphis gossypii* Glover (Hemiptera: Aphididae) causes both direct and indirect damage. Direct damage includes feeding injuries to foliage [8, 9]. Indirect damage occurs through the excretion of honeydew, which promotes the growth of sooty mold. Honeydew production by aphids plays a broader ecological role in agroecosystems by mediating interactions with other arthropods and influencing multitrophic relationships, thereby affecting pest population dynamics and biological control processes [10]. Additionally, many aphid species serve as vectors for potyviruses [11, 12, 13]. Wingless aphids develop faster than winged forms and exhibit adaptations that enhance fecundity, making them more difficult to control [14]. The natural enemy selected for this study was *Chrysoperla carnea* (Stephens) (Neuroptera: Chrysopidae), a cosmopolitan and polyphagous predator commonly found in both natural and agricultural ecosystems worldwide. It is also commercially available in many countries for the biological control of aphids and other soft‐bodied herbivorous insects in greenhouse crops [15]. Recent studies have demonstrated that the developmental rate, survival, and reproductive performance of *Chrysoperla carnea* are strongly influenced by aphid prey quality, with wingless aphid morphs providing superior nutritional value and significantly enhancing predator fitness and fecundity, highlighting the ecological adaptability and effectiveness of this predator in aphid‐based biological control systems [16].

Thrips, in contrast, feed by piercing plant cells and extracting their contents, leading to the formation of small, whitish streaks on leaves and fruit, as well as deformities in developing fruit [17, 18]. In addition to direct feeding damage and associated yield loss, certain thrips species are known vectors of Tomato Spotted Wilt Virus (TSWV), a significant pathogen affecting both pepper and tomato crops [19, 20, 21]. Additionally, several species of the anthocorid bug *Orius* have been studied for their effectiveness in managing thrips populations in protected sweet pepper cultivation [7]. These predators, commonly found in flowers—also a preferred habitat for thrips—can prey on both adult and immature stages. However, their small size limits their ability to reach concealed areas where immature thrips often reside [22]. As alternative measures to chemical control, several strategies stand out: managing host plants located within and/or along the edges of the crop to ensure that their growth cycle does not coincide with periods of heightened crop susceptibility, thereby avoiding severe infestations during critical growth stages; using blue and yellow sticky traps to monitor and reduce pest populations; and applying biological control methods, such as introducing predators like *Orius* spp [23].

In addition to the resistance issues that may be intensified by climate change [24], pesticides are known to have significant negative impacts on both the environment and human health [25]. However, eliminating the use of pesticides is not as straightforward as one might hope. Insect development and reproduction are tightly regulated by endocrine and metabolic pathways, particularly juvenile hormone biosynthesis, making these physiological processes attractive targets for developing alternative and more selective pest management strategies [26]. They are not easily replaced by alternative methods that can match their effectiveness in controlling pests and diseases, which is essential for ensuring the high crop yields that farmers depend on for economic survival and that consumers rely on for their daily intake of nutrients and other essential goods [27]. Systemic acquired resistance (SAR) is a relatively recent concept in pest management and has shown limited effectiveness against sucking insect pests [28]. However, SAR activators can be effectively integrated into Integrated Pest Management (IPM) programs when applied at appropriate concentrations and tailored to specific plant growth stages [29, 30, 31]. The main goal of induced resistance is to provide broad‐spectrum, long‐lasting protection [32, 33]. Resistance inducers are regarded as environmentally friendly alternatives, as they have demonstrated significant potential in reducing the severity of major plant diseases [34, 35, 36, 37, 38].

Phosphite, a derivative of phosphorous acid (H_3_PO_3_), is commonly referred to as Phi, representing the reduced form of inorganic phosphate. It should not be confused with phosphate ions such as HPO_4_^2^^−^ and H_2_PO_4_^−^. Among the most widely used phosphite compounds is potassium phosphonate [24]. Due to its systemic mobility between leaves and roots, Phi functions as a plant defense inducer, with well‐documented stimulatory effects. For example, phosphite (Phi) has been reported to activate genes associated with the jasmonic acid (JA) and ethylene (ET) signaling pathways, thereby enhancing the production of phytoalexins and chitinases and promoting cell wall reinforcement [35, 36, 39, 40]. As a promising alternative to conventional pesticides, phosphite offers the advantage of minimal risk to human health and the environment [41].

Effective Microorganisms (EM) are a liquid mixture of approximately 80 beneficial and non‐pathogenic microbes, including both aerobic and anaerobic types, that coexist harmoniously and positively impact the environment [42, 43]. These microbes work synergistically to produce substances that can suppress diseases [44] and have potential in insect control due to their ability to generate insect‐repelling esterases, defensive enzymes, and hydrolytic acids [45, 46]. EM also promotes induced systemic resistance (ISR) in plants, enhancing their natural defense mechanisms [38, 47]. Additionally, EM produces uncommon soil compounds such as lactic acid, acetic acid, amino acids, malic acid, and vitamins, which are readily absorbed by plants and contribute to improved growth [48].

Salicylic acid (SA) is a naturally occurring phenolic compound and plant hormone that plays a vital role in plant responses to various abiotic stresses and pest attacks [49]. Among plant resistance inducers, those that activate Systemic Acquired Resistance (SAR)—a defense mechanism primarily mediated by SA—are considered the most effective [27]. SA is also closely associated with several physiological processes that support plant growth and development [50]. Moreover, it has been shown to activate defense‐related genes, resulting in the synthesis and accumulation of antioxidant enzymes, steroidal glycoalkaloids, and volatile organic compounds (VOCs). These VOCs help enhance indirect plant defenses by attracting natural enemies of herbivorous insects [51, 52]. Additionally, the exogenous application of SA has been found to reduce caterpillar feeding and delay the development of insect resistance [53].

Imidacloprid is a first‐generation, chlorinated neonicotinoid insecticide that exhibits multiple modes of action, including contact toxicity, ingestion poisoning, and internal absorption through inhalation [54]. It is effective against various pests, particularly thrips and other insects with piercing‐sucking mouthparts. The primary mechanism behind imidacloprid's efficacy lies in its ability to mimic acetylcholine, thereby disrupting normal neural activity in insects. This leads to prolonged overstimulation of the nervous system and ultimately, insect death [55, 56]. However, the application of imidacloprid in agricultural ecosystems can also impact non‐target organisms, potentially contaminating soil and water resources and disrupting the food web, which may compromise the overall ecological balance [57]. Previous studies have explored whether imidacloprid use poses a threat to the pest control capabilities of *Orius similis* [12, 58]. In contrast, dinotefuran is a third‐generation neonicotinoid insecticide known for its rapid action, high efficacy, long‐lasting effects, and broad‐spectrum activity, all while exhibiting low phytotoxicity [59]. Although pests such as whiteflies may develop resistance to imidacloprid, dinotefuran remains effective for sustained population control [60].

The hypothesis proposed that activating plant defenses using the tested agents could influence insect development, reproduction, and feeding behavior on pepper plants. Accordingly, this study aimed to evaluate plant parameters—such as total chlorophyll and total protein content—as indicators of enhanced plant growth. These improvements may reinforce the plant's internal defense mechanisms, thereby reducing pest populations and ultimately increasing sweet pepper yield. Additionally, the study investigated whether these treatments could simultaneously suppress herbivorous insect populations and enhance sweet pepper productivity.

## 2 Materials and Methods

### 2.1 Study Site

This research was conducted in a greenhouse setting in Kafr Al Battikh city, located in Damietta province (31°2500′00″N, 31°49′1700″E), Egypt, over two growing seasons in 2022 and 2023.

### 2.2 The Tested Formulations

The effective microorganisms (EMs) used in this study were obtained from the Ministry of Agriculture in Cairo, Egypt. The EM formulation contained a variety of beneficial microorganisms, which were cultured in specific media and produced under the supervision of the Japanese EMs Research Organization in Egypt [45]. The microbial consortium comprised photosynthetic bacteria (*Rhodobacter sphaeroides* and *Rhodopseudomonas palustris*), lactic acid bacteria (*Lactobacillus casei*, *Lactobacillus plantarum*, and *Streptococcus lactis*), actinomycetes (*Streptomyces griseus* and *Streptomyces albus*), yeasts (*Candida utilis* and *Saccharomyces cerevisiae*), and fermenting fungi (*Mucor hiemalis* and *Aspergillus oryzae*), as previously documented [61]. The formulation had a pH of 3.00 and was stored in a refrigerator at 0°C. Prior to use, it was diluted in water at a concentration of 5 mL/L. Neonicotinoids included: Imidacloprid (IMI) (Admire 20% SL) was purchased from BAYER and applied at a dosage of 119.05 mL/ha. Dinotefuran (Oshin 20% SG) was obtained from the Mitsui Chemical Company and applied at a dosage of 476.19 mL/ha. Potassium phosphite (PK), branded as Naturephos (58% P_2_O_5_ and 32% K_2_O), was sourced from Daymasa Company, Zaragoza, Spain, and used at a dosage of 1 g/L. Salicylic acid (SA) (phenolic acid), with a purity of 99.99%, was purchased from Al‐Gomhoreya Chemical Company, El Geish St., Tanta, Egypt, and applied at a dosage of 2 mm/L.

### 2.3 The Experimental Procedure

The experimental area of the greenhouse covered approximately 4200 m^2^. Sweet pepper seedlings (var. TOP STAR; Takii and Co., Ltd., Kyoto, Japan) were transplanted on 22 September 2022 and 29 September 2023. All standard agricultural practices were followed, and treatments were arranged in a randomized complete block design. Five treatments were evaluated: imidacloprid (IMI; synthetic insecticide), potassium phosphite (PK), effective microorganisms (EMs), salicylic acid (SA), and a water‐sprayed untreated check (control). Treatment plots were sprayed with the respective agents 27 days after planting, whereas control plots received water only. When insect infestation reached the predefined economic threshold of 3.00% (45 days after planting), dinotefuran was applied at the recommended rate to all treated plots, while the water‐sprayed untreated check (control) remained unsprayed. Dinotefuran was used as a standardized rescue spray to prevent crop loss and to simulate commercial integrated pest management (IPM) conditions; therefore, pest and predator responses are interpreted as treatment effects under dinotefuran overlap rather than as insecticide‐free control. Each treatment and the water‐sprayed untreated check (control) were replicated three times, with each replicate consisting of 150 plants occupying an area of 75 m^2^. All agricultural practices were conducted to reflect commercial sweet pepper production conditions.

### 2.4 Laboratory Determination of Plant Parameters Affected by Microbial and Chemical Agents

After the application of microbial and chemical agents for 4 days, five plants were randomly selected from each replicate and transferred to the laboratory. Dried leaf samples were subsequently analyzed for photosynthetic pigment content. Total chlorophyll content was determined using the equations previously described [62]. Nitrogen (N) and phosphorus (P) contents were measured according to established methods [63], while potassium (K) content was estimated following a standard procedure [64]. Phenolic compounds were quantified using a previously described technique [65]. Finally, total protein content was determined using an established method [66].

### 2.5 Field and Laboratory Inspection of the Tested Insects

Twenty‐four plant leaves were randomly inspected at three different levels of the plant canopy (8 leaves from the upper, 8 from the middle, and 8 from the lower canopy) for each replicate. Insect adults were counted and recorded in the field at 6:00 am, one day before treatment, and 1, 3, and 7 days after the application of dinotefuran. For insect count determination, the same set of twenty‐four leaves from each replicate was collected at the three plant levels—upper, middle, and lower canopy—on the specified days. The leaves were placed individually into plastic bags and transported directly to the laboratory. Once in the lab, the leaves were examined under a binocular microscope, and the number of insects present was counted and recorded.

The reduction percentage was calculated using the equation described previously [67], as follows:

$$\mathrm{Reduction}\;\left(\%\right)=100\;\left[1-\frac{B\times A^{\prime }}{B^{\prime }\times A}\right]$$

where:

B = Number of individuals in the treated samples after spraying the tested agents.

B′ = Number of individuals in the treated sample before spraying the tested agents.

A = Number of individuals in the untreated check samples (control) after spraying with water.

A′ = Number of individuals in the untreated check samples (control) before spraying with water.

### 2.6 Statistical Analysis

Data were analyzed in RStudio (version 4.4.1). Normality was assessed using the Shapiro–Wilk test, and homogeneity of variances was evaluated using Levene's test where applicable. Parameters that met normality and homogeneity assumptions were analyzed using one‐way analysis of variance (ANOVA). Physiological, biochemical, plant growth, and fruit yield parameters of sweet pepper (Tables S1 and S2) were measured once per experimental unit using three independent replicate plots per treatment and were analyzed as independent observations.

Pest reduction data for *Aphis gossypii* (Table S3), which involved repeated observations across instars (first, third, and seventh) and seasons, were analyzed using linear mixed‐effects models, with treatment included as a fixed factor and instar and season treated as random factors to account for within‐unit correlation.

For datasets that did not meet normality assumptions, including reduction percentages of *Thrips tabaci* and the predator species *Chrysoperla carnea* and *Orius insidiosus* (Tables S4–S6), treatment effects were evaluated using non‐parametric Kruskal–Wallis tests, followed by appropriate post hoc comparisons where applicable. Statistical significance was determined at α = 0.05.

## 3 Results

### 3.1 Effect of Tested Agents on Sweet Pepper Parameters

Data presented in Table S1 and Figure 1 indicate that the tested materials significantly influenced the physiological and biochemical parameters of sweet pepper plants. Among the evaluated treatments, salicylic acid (SA) exerted the most pronounced positive effect on total chlorophyll content, followed by potassium phosphite (PK) and effective microorganisms (EMs). In contrast, imidacloprid (IMI) significantly reduced total chlorophyll levels compared with the water‐sprayed untreated check (control). Salicylic acid was also the most effective treatment in enhancing macronutrient concentrations, including nitrogen (N), phosphorus (P), and potassium (K), in plant tissues. All treatments resulted in increased levels of these nutrients relative to the control. Total phenolic content was significantly elevated by SA and PK, whereas all tested treatments increased total protein content in both leaves and fruits compared with the control, with SA producing the greatest enhancement. All physiological and biochemical parameters were measured once per experimental unit using three independent replicates per treatment (*n* = 3). Variables meeting normality and homogeneity assumptions were analyzed using one‐way ANOVA, which revealed significant treatment effects on total chlorophyll content and macronutrient concentrations, particularly nitrogen (*p* < 0.001). Parameters that did not meet normality assumptions, including total phenolic content and protein levels, were analyzed using the Kruskal–Wallis test followed by Dunn's post hoc comparisons (*p* < 0.05). As shown in Table S1 and Figure 1, SA treatment consistently resulted in the highest chlorophyll content, nitrogen concentration, and protein levels compared with the water‐sprayed untreated check (control), while PK, EMs, and IMI produced significant but comparatively lower improvements.

FIGURE 1Effects of microbial and chemical agents on physiological and biochemical parameters of sweet pepper plants. Panels show (A) total chlorophyll content, (B) nitrogen, (C) phosphorus, (D) potassium, (E) total phenolic content, (F) leaf protein content, and (G) fruit protein content. All parameters were measured once per experimental unit at the end of the experiment. Data are presented as mean values from three independent replicates per treatment (*n* = 3). Treatment effects were analyzed using one‐way ANOVA for normally distributed variables and the Kruskal–Wallis test with appropriate post hoc comparisons for non‐normally distributed variables (α = 0.05). Different letters indicate statistically significant differences among treatments.

**Figure:**
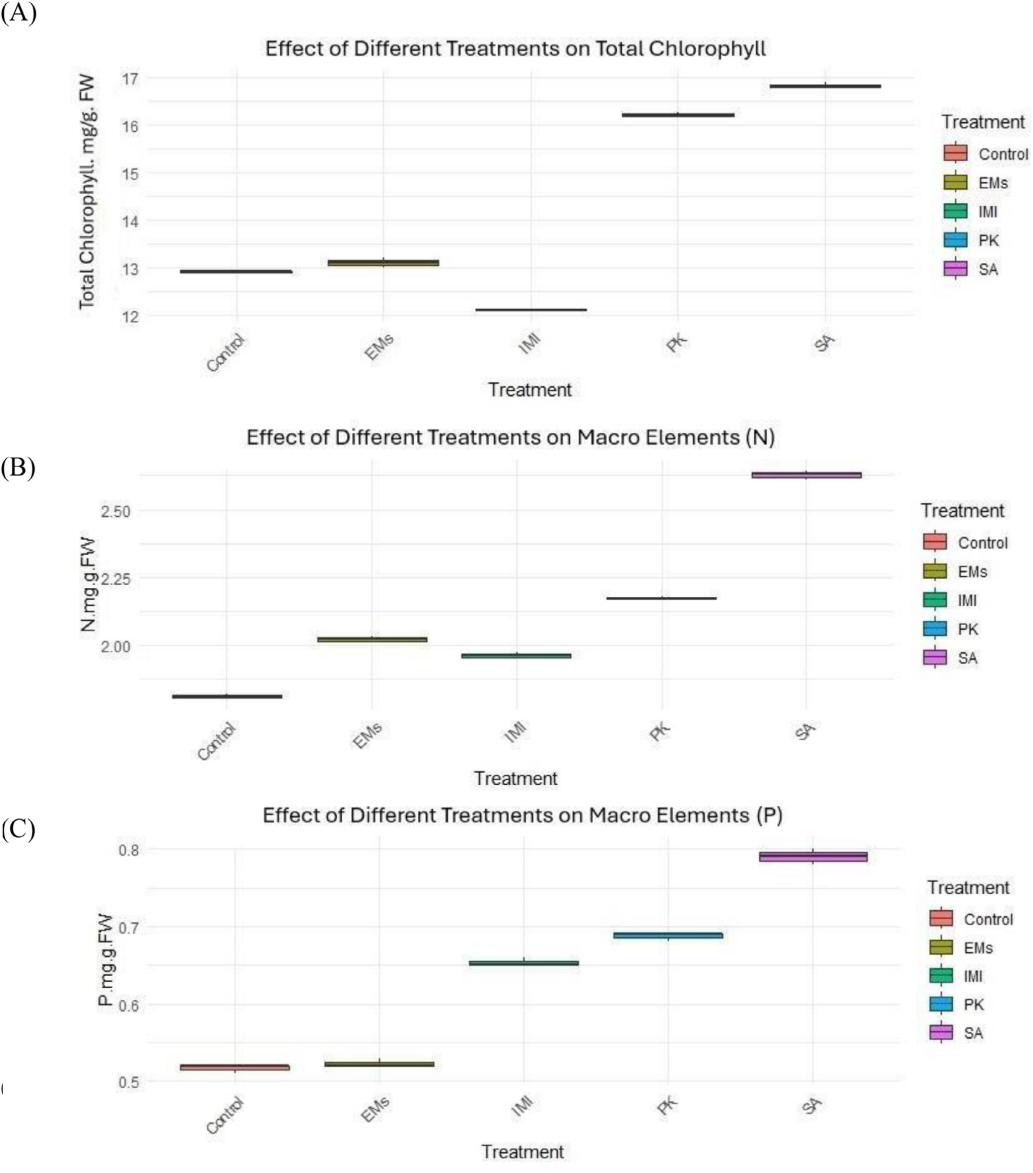

**Figure:**
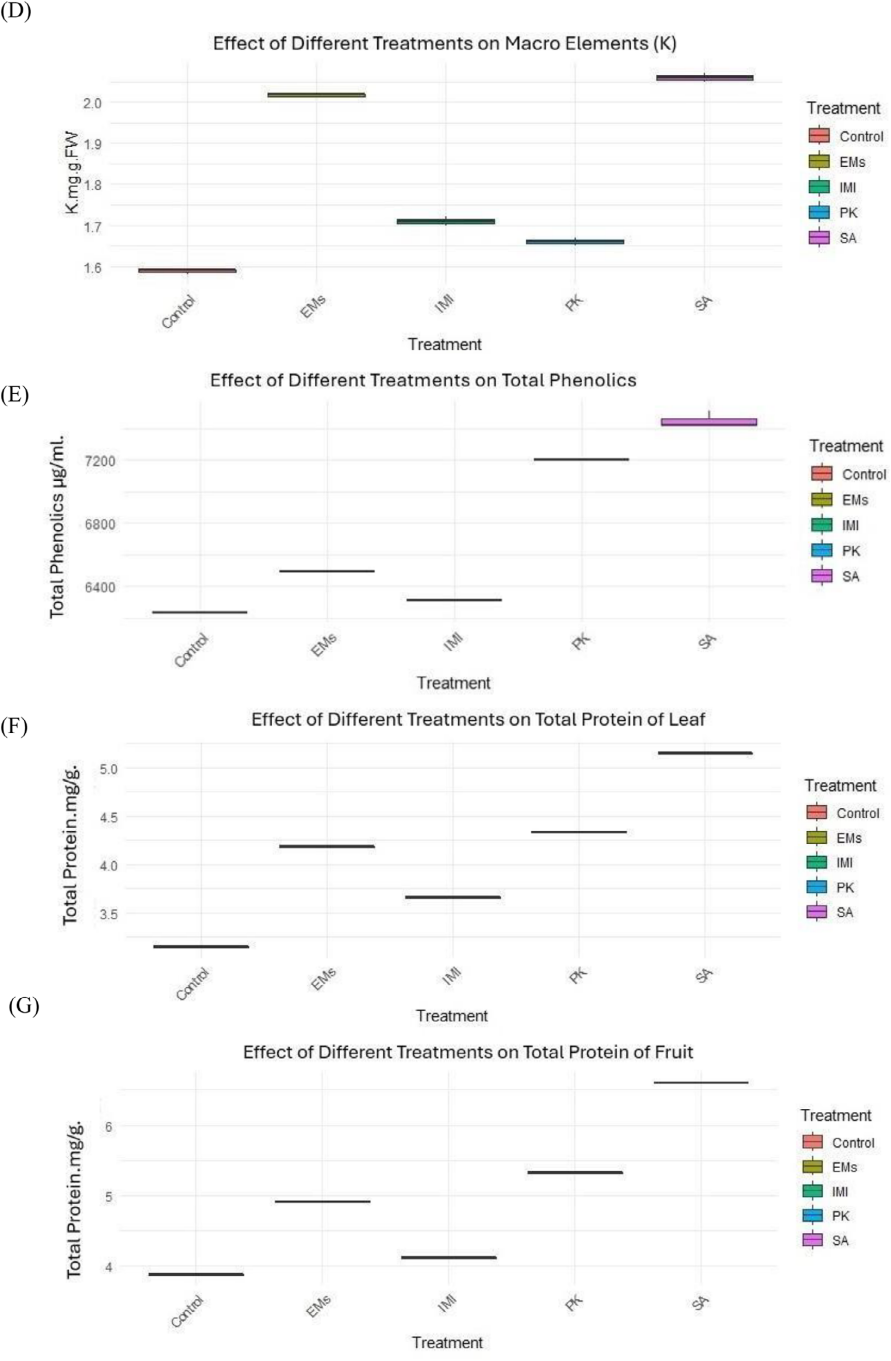

Data presented in Table S2 and Figure 2 show that the applied treatments significantly influenced pepper growth and fruit yield parameters. Among the tested agents, salicylic acid (SA) was the most effective in improving vegetative growth traits, including plant height, number of leaves, number of branches per plant, and plant fresh and dry weights. In contrast, potassium phosphite (PK) exerted the strongest positive effect on fruit characteristics, including fruit number, length, and diameter, followed by effective microorganisms (EMs). Plant growth and fruit yield parameters were recorded once per experimental unit using three independent replicates per treatment (*n* = 3). Normality assumptions were met for most variables (Shapiro–Wilk test, *p* > 0.05), with the exception of plant fresh weight, while homogeneity of variances was confirmed using Levene's test (*p* > 0.05). Multivariate analysis revealed a significant overall effect of treatment on plant growth and fruit yield parameters (*p* < 0.001). Subsequent univariate analyses indicated significant treatment effects on all measured traits (*p* < 0.001). As shown in Table S2 and Figure 2, PK significantly increased fruit number and fruit size compared with the water‐sprayed untreated check (control), whereas SA predominantly enhanced vegetative growth parameters, particularly plant height and branching.

FIGURE 2Effects of microbial and chemical agents on plant growth and fruit yield parameters of sweet pepper. Panels show plant height, number of leaves, number of branches, plant fresh and dry weight, number of fruits per plant, fruit length, and fruit diameter. All parameters were measured once per experimental unit at the end of the growing season. Data are presented as mean values calculated from three independent replicates per treatment (*n* = 3). Treatment effects were assessed using multivariate analysis followed by univariate analyses; normally distributed variables were analyzed using one‐way ANOVA, whereas non‐normally distributed variables were analyzed using the Kruskal–Wallis test. Different letters above bars indicate statistically significant differences among treatments.

**Figure:**
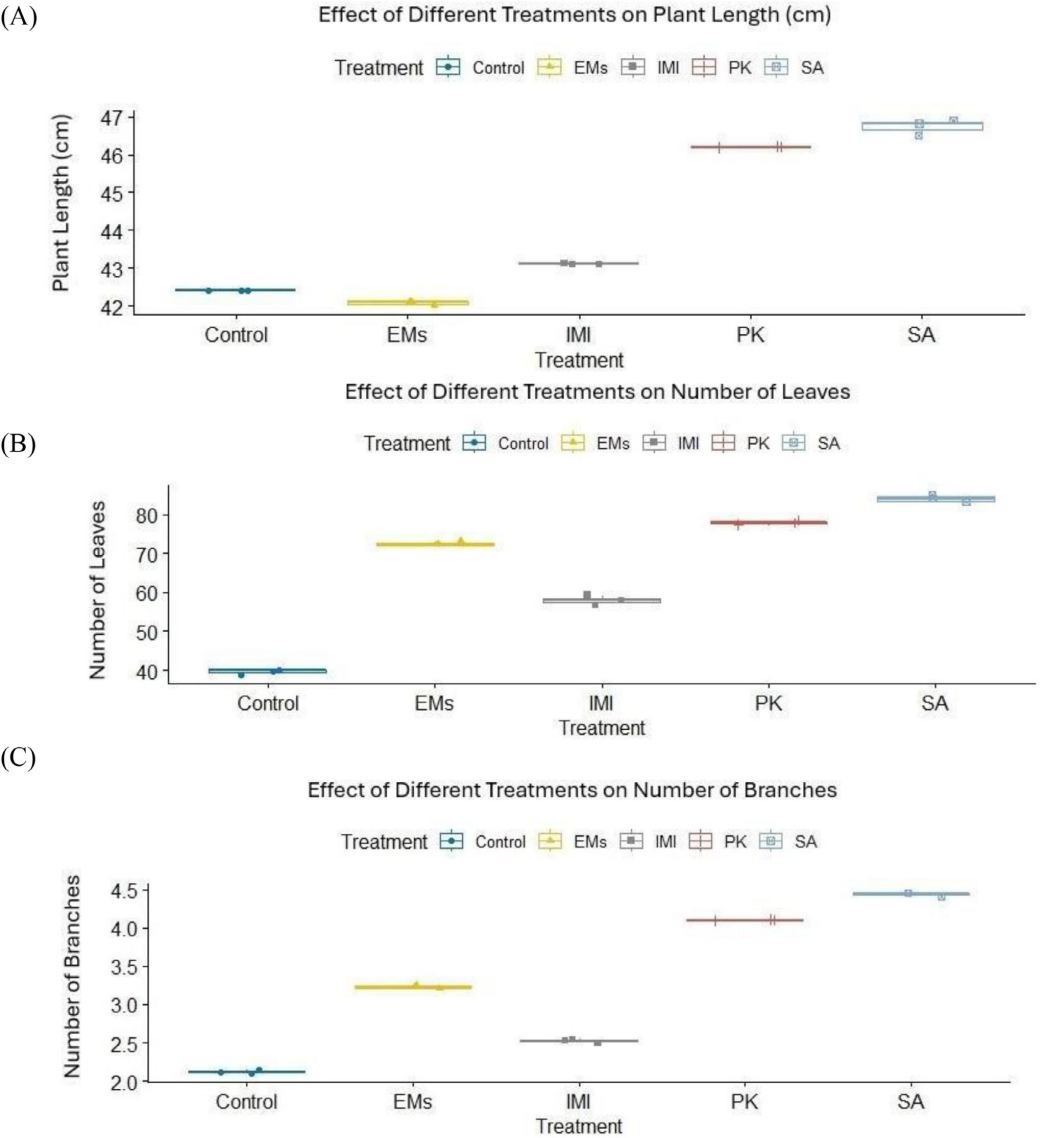

**Figure:**
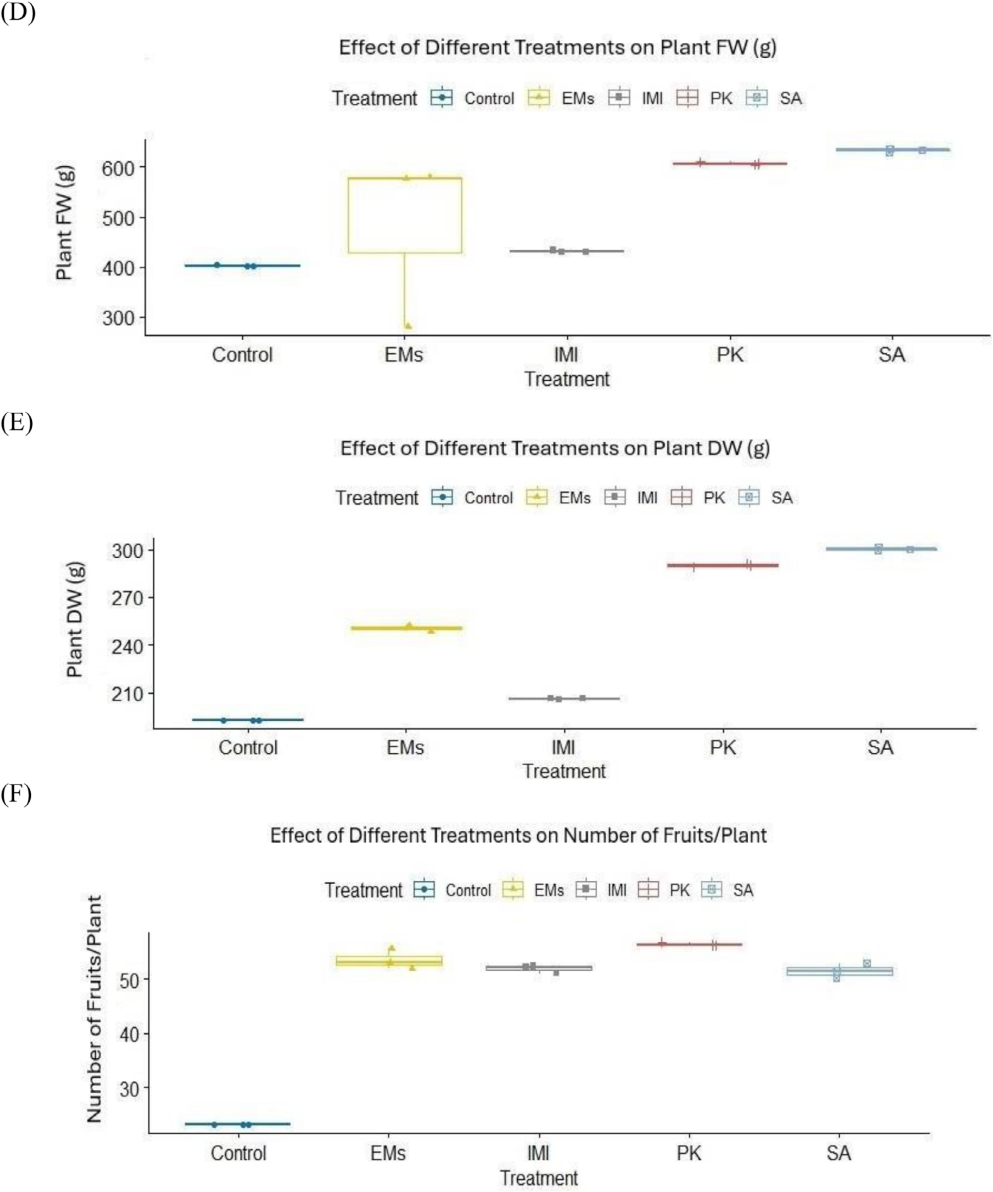

**Figure:**
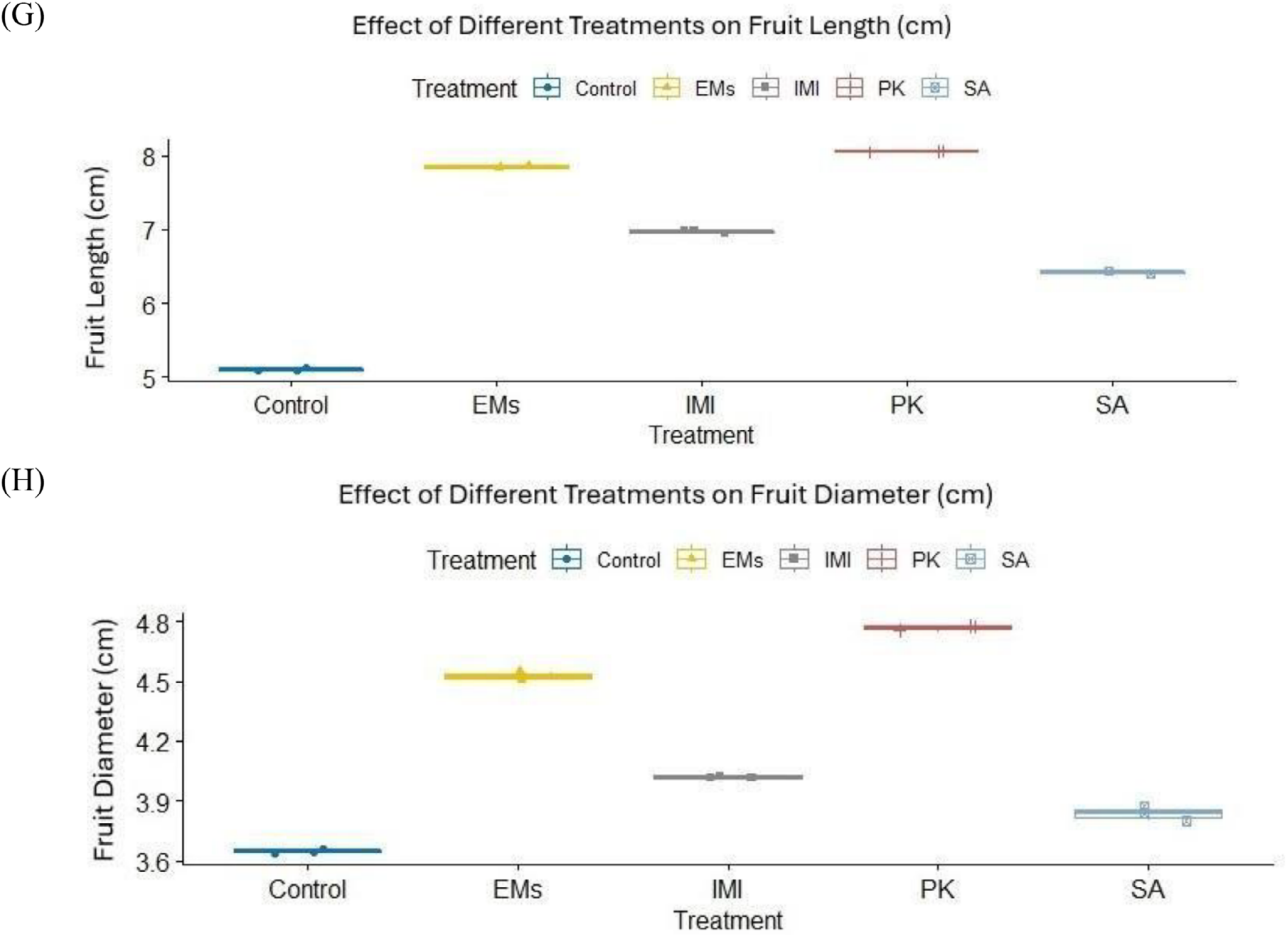

### 3.2 Effect of Tested Agents on Target Insects during the 2022 and 2023 Seasons

**FIGURE 3:**
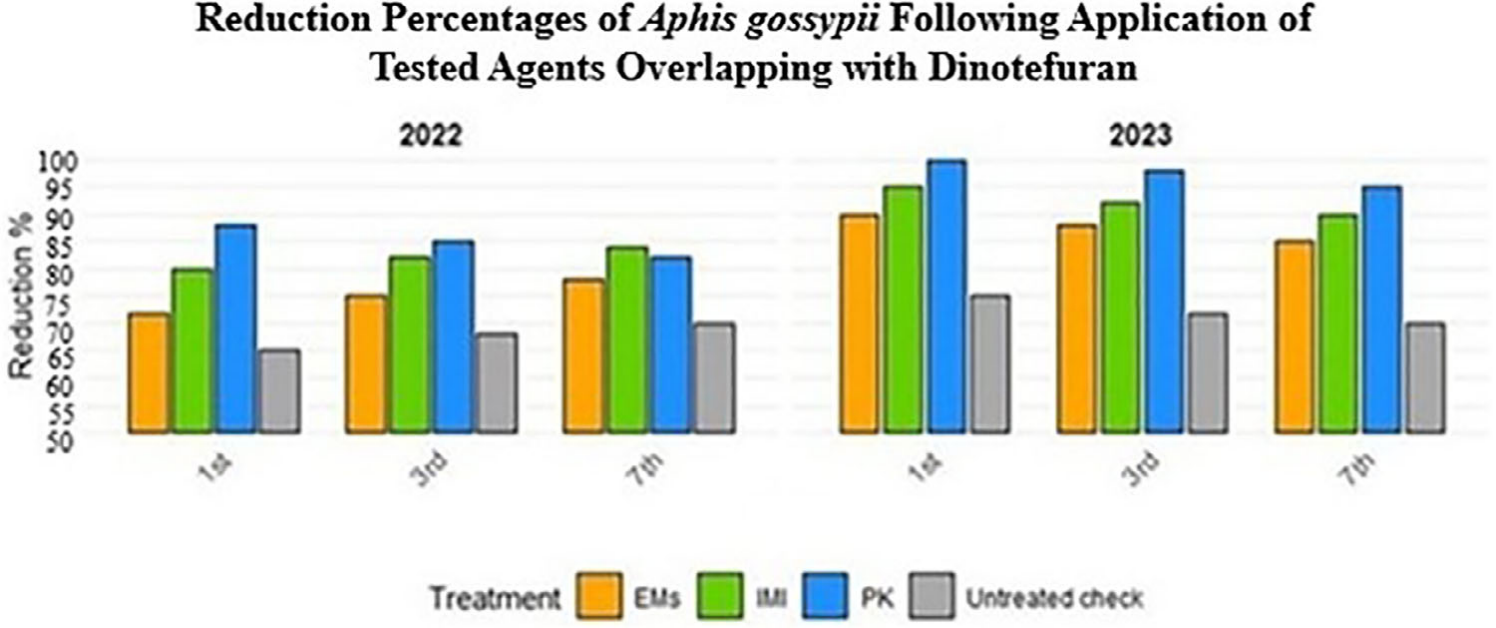
Reduction percentages of *Aphis gossypii* following application of tested agents overlapping with dinotefuran across different instars (first, third, and seventh) during the 2022 and 2023 seasons. Data represent repeated observations collected from the same experimental plots and are presented as mean values from three independent replicate plots per treatment (*n* = 3). Treatment effects were analyzed using a linear mixed‐effects model to account for repeated measurements, followed by Tukey's post hoc comparisons (α = 0.05). Different letters indicate statistically significant differences among treatments.

Data presented in Table S3 and Figure 3 indicate overlapping indirect effects of the tested agents on the target insect pest *Aphis gossypii* under greenhouse conditions during the 2022 and 2023 seasons, with treatment effects interpreted under dinotefuran overlap. All tested agents resulted in significantly greater reductions in aphid populations compared with the water‐sprayed untreated check (control). Among the evaluated treatments, potassium phosphite (PK) was the most effective against *A. gossypii* in both seasons, achieving mean reduction rates of 89.02% and 88.71% in 2022 and 2023, respectively. Reduction percentages of *A. gossypii* were calculated for different instars (first, third, and seventh) across two growing seasons using repeated observations from the same experimental plots, with three independent replicate plots per treatment. Normality of model residuals was confirmed using the Shapiro–Wilk test (*p* = 0.869). Treatment effects on aphid reduction were evaluated using a linear mixed‐effects model, with treatment included as a fixed factor and season and instar treated as random factors to account for repeated measurements within experimental units. The analysis revealed a highly significant effect of treatment on aphid reduction (*p* < 0.001). Overall, phosphite (PK) consistently resulted in the highest reduction of *A. gossypii* populations across instars and seasons.

**FIGURE 4:**
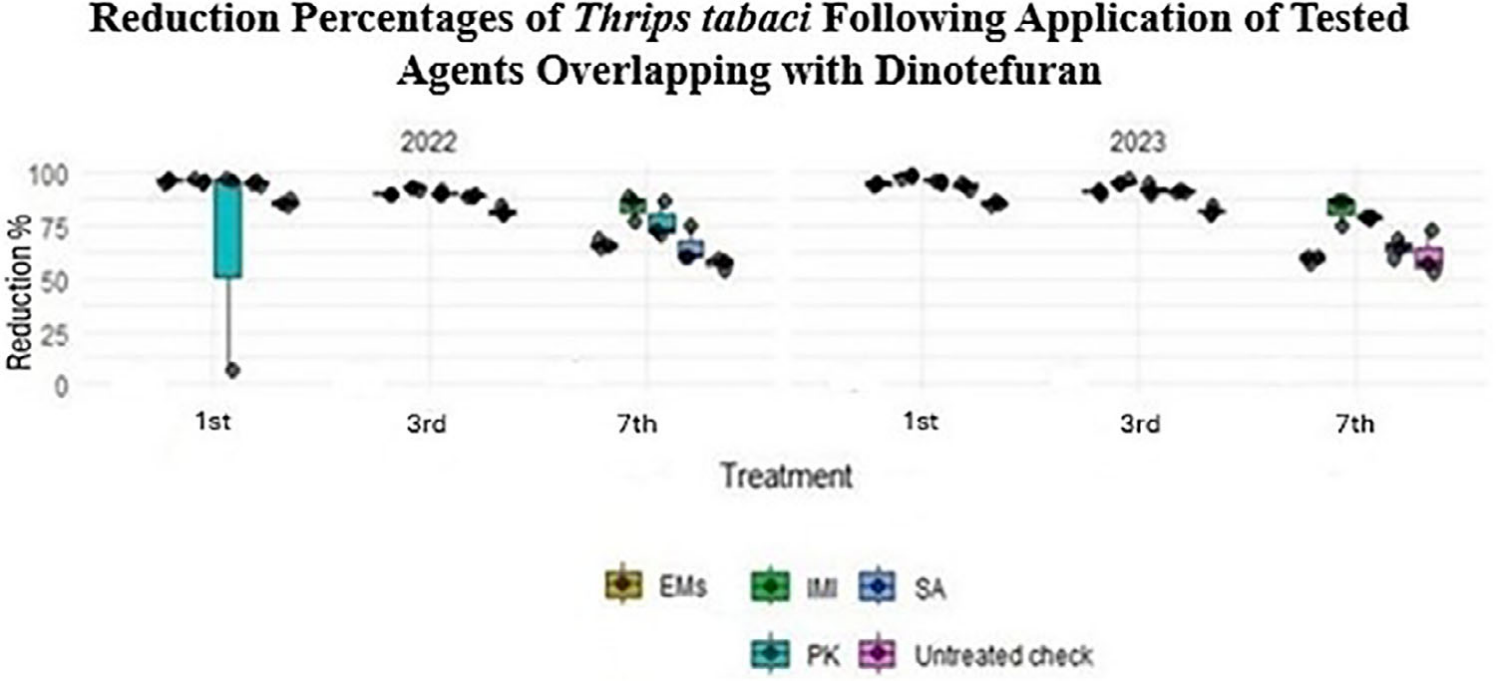
Reduction percentages of *Thrips tabaci* following application of tested agents overlapping with dinotefuran across different instars (first, third, and seventh) during the 2022 and 2023 seasons. Data represent repeated observations collected from the same experimental plots and are presented as mean values from three independent replicate plots per treatment (*n* = 3). Treatment effects were evaluated using the Kruskal–Wallis test (α = 0.05). Different letters indicate statistically significant differences among treatments.

Conversely, data presented in Table S4 and Figure 4 showed that imidacloprid (IMI) exhibited the highest efficacy against *Thrips tabaci* across both growing seasons, achieving mean reduction values of 89.02% and 88.71% in 2022 and 2023, respectively, when compared with the water‐sprayed untreated check (control). Reduction percentages of *T. tabaci* were assessed across different instars (first, third, and seventh) using repeated observations from the same experimental plots over two growing seasons, with three independent replicate plots per treatment. Normality testing using the Shapiro–Wilk test indicated that the data did not meet normality assumptions (*p* < 0.05). Consequently, treatment effects were analyzed using the Kruskal–Wallis test, which revealed significant differences among treatments (*p* < 0.001). Overall, across instars and seasons, IMI consistently resulted in the greatest reduction of *T. tabaci* populations.

### 3.3 Effect of Tested Agents on the Predators during the 2022 and 2023 Seasons

**FIGURE 5:**
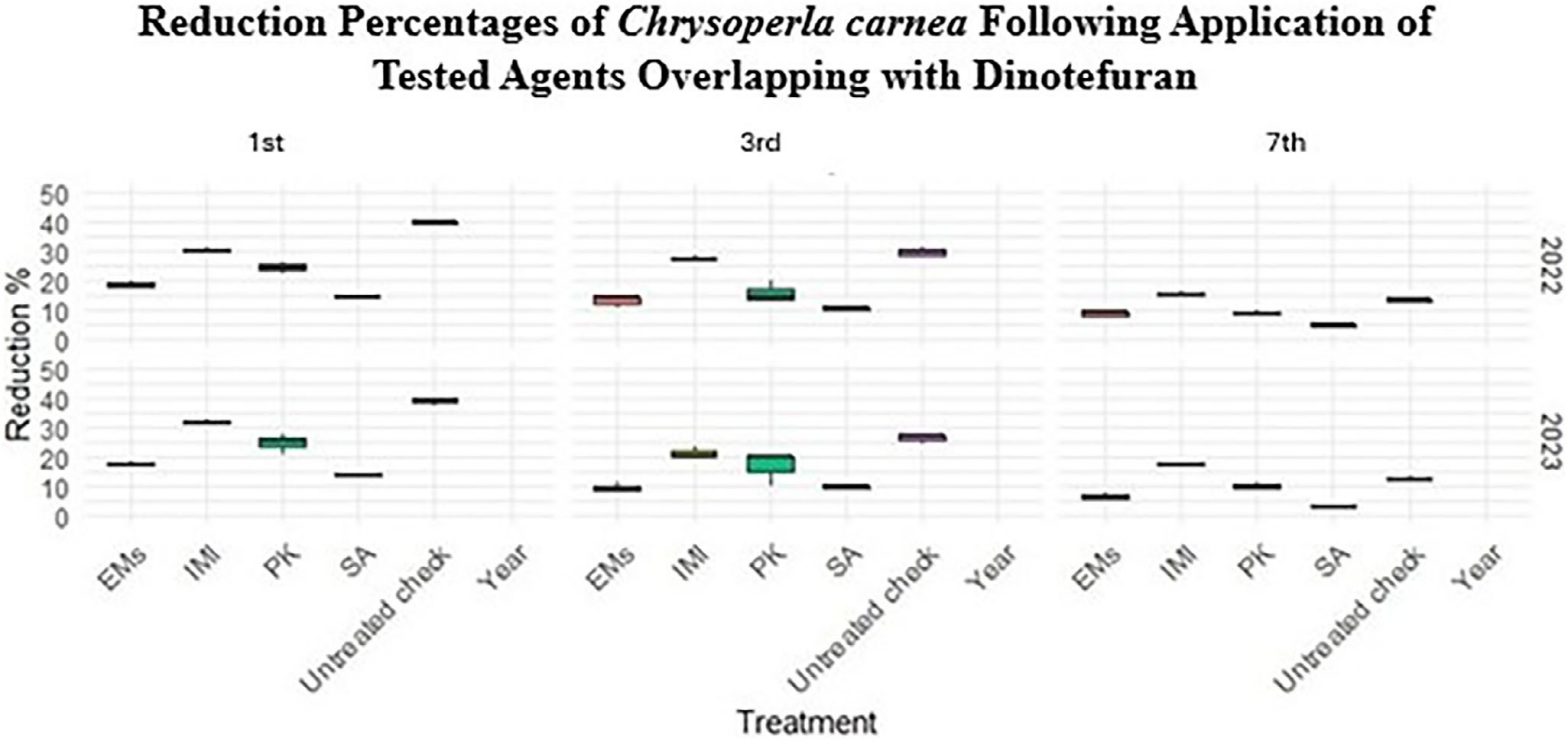
Reduction percentages of *Chrysoperla carnea* across different instars (first, third, and seventh) following application of tested agents overlapping with dinotefuran during the 2022 and 2023 seasons. Data represent repeated observations collected from the same experimental plots and are presented as mean values from three independent replicate plots per treatment (*n* = 3). Treatment effects were evaluated using the Kruskal–Wallis test with pairwise Wilcoxon rank‐sum comparisons and Holm correction (α = 0.05). Different letters indicate statistically significant differences among treatments.

**FIGURE 6:**
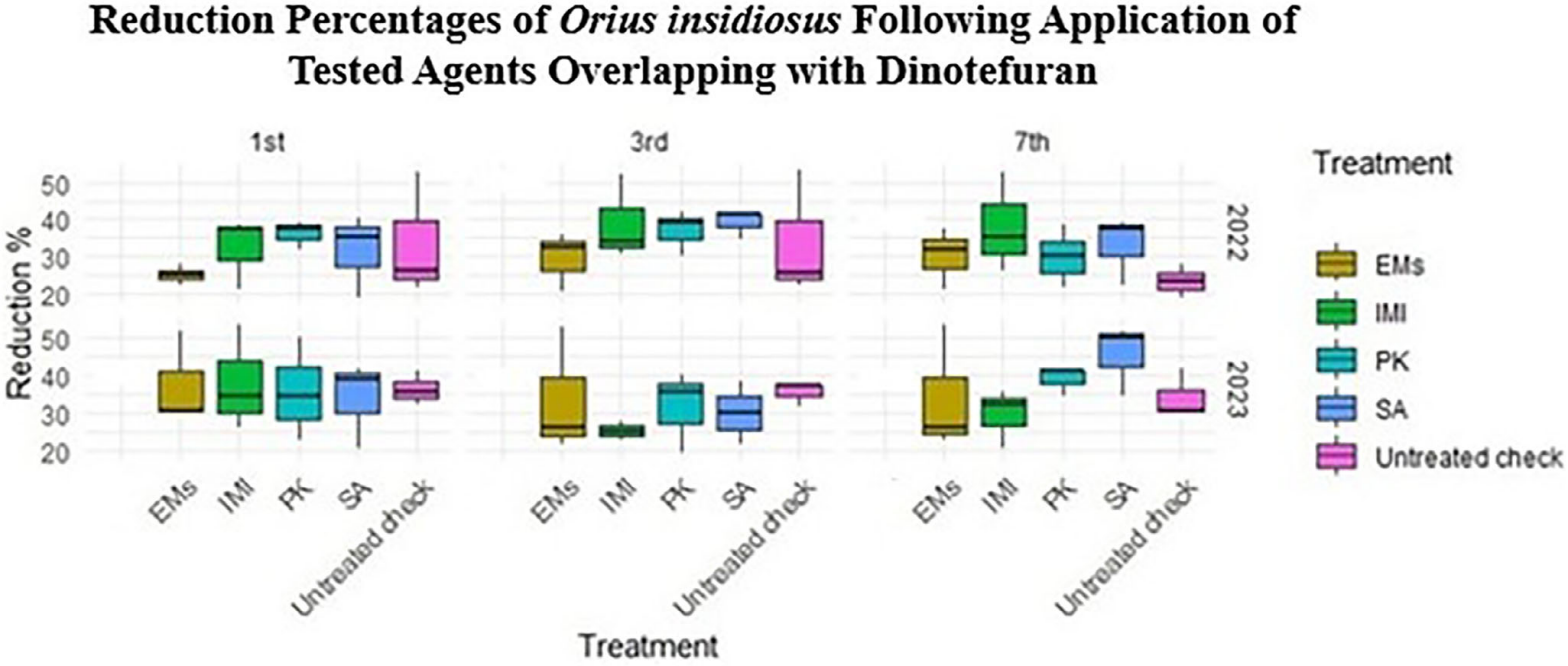
Reduction percentages of *Orius insidiosus* across different instars (first, third, and seventh) following application of tested agents overlapping with dinotefuran during the 2022 and 2023 seasons. Data represent repeated observations collected from the same experimental plots and are presented as mean values from three independent replicate plots per treatment (*n* = 3). Treatment effects were evaluated using the Kruskal–Wallis test (α = 0.05). No significant differences among treatments were detected.

Data presented in Table S5 and Figure 5 reveal overlapping indirect effects of the tested agents on populations of the predator *Chrysoperla carnea* during the 2022 and 2023 seasons under dinotefuran overlap. All treatments resulted in greater reductions compared with the water‐sprayed untreated check (control); however, non‐conventional treatments (SA, PK, and EMs) exhibited greater selectivity toward predator populations, showing significantly lower reduction percentages than the synthetic insecticide imidacloprid (IMI). This indicates a comparatively reduced negative impact of these treatments on beneficial predators, particularly when compared with IMI. Reduction percentages of *C. carnea* were assessed across different instars (first, third, and seventh) over two growing seasons using repeated observations from the same experimental plots, with three independent replicate plots per treatment (*n* = 3). Normality testing using the Shapiro–Wilk test indicated non‐normal data distribution (*p* < 0.001). Consequently, treatment effects were analyzed using the Kruskal–Wallis test, which revealed significant differences among treatments (*p* < 0.001). Across instars and seasons, IMI consistently resulted in the greatest reduction of *C. carnea* populations. In contrast, data presented in Table S6 and Figure 6 show that reduction percentages of *Orius insidiosus* did not differ significantly among treatments across the 2022 and 2023 growing seasons. Reduction percentages were assessed for different instars (first, third, and seventh) using repeated observations from the same experimental plots, with three independent replicate plots per treatment (*n* = 3). Normality testing using the Shapiro–Wilk test indicated non‐normal data distribution (p = 0.001); therefore, treatment effects were evaluated using the Kruskal–Wallis test, which revealed no significant differences among treatments (*p* = 0.161).

## 4 Discussion

Insecticides are an essential and widely used tool in agricultural production. However, their improper application can negatively impact the food chain and ecosystem [12]. *Aphis gossypii* and *Thrips tabaci* have developed resistance to several major families of synthetic insecticides [68]. To promote sustainable agriculture, it is crucial to reduce insecticide use. One promising strategy within Integrated Pest Management (IPM) is the use of elicitors—plant strengtheners that can induce herbivore resistance and enhance biological control of pests [69]. Pretreating plants with various chemical inducers can trigger resistance mechanisms, offering protection against insect attacks. In response to herbivory, plants activate a range of inducible defense mechanisms [70]. The findings of the current study showed that foliar application of IMI, PK, EMs, and SA to sweet pepper plants on the 27th day after planting significantly reduced aphid and thrips infestations compared to the control treatment. Because dinotefuran was applied as a rescue intervention once infestation reached 3%, the present study evaluates treatment performance under dinotefuran overlap and does not represent a fully insecticide‐free test of induced resistance.

Chlorophyll content was measured to evaluate the enhancing effects of the tested agents on pepper plants. Chlorophyll is a naturally occurring pigment that absorbs light energy to facilitate photosynthesis. A better understanding of chlorophyll pigment content is expected to lead to improved methods for assessing plant responses to environmental stresses [71]. Recent studies suggest that salicylic acid (SA) plays a key role in regulating photosynthesis, as it influences both leaf and chloroplast structure [72]. In the present study, SA was the most effective treatment for increasing total chlorophyll content, followed by phosphite. These results are in agreement with previous findings [73, 74, 75]. Salicylic acid (SA) application has been shown to improve the chlorophyll fluorescence ratio in cucumber (*Cucumis sativus* L.) [73]. In addition, SA functions as an important antioxidant by enhancing the activities of peroxidase, superoxide dismutase, and catalase, thereby reducing reactive oxygen species (ROS) and promoting chlorophyll synthesis [74]. Furthermore, SA, as a phytohormone, is widely used to mitigate the adverse effects of salt stress in plants [75]. In contrast, treatment with imidacloprid (IMI) resulted in a reduction in total chlorophyll content compared with the control. This decrease may be attributed to the activation of proteolytic enzymes such as chlorophyllase, which is responsible for chlorophyll degradation [76]. Additionally, the decline in chlorophyll may be linked to increased lipid peroxidation and higher levels of ROS generation [77].

Reactive oxygen species (ROS) are crucial signaling molecules involved in various key physiological processes in plants. However, they are also toxic by‐products of aerobic metabolism. ROS have been widely used as indicators to assess the degree of oxidative damage in fruits and vegetables [78]. Plant phenolic compounds are known to act as reducing agents, free radical scavengers, and chelators of pro‐oxidant catalytic metals, thereby preventing lipid peroxidation [65]. Salicylic acid (SA) has been identified as an effective treatment for increasing phenolic content. This finding is supported by previous studies showing that exogenous application of salicylic acid (SA) increases total phenolic content in plants [79]. Similarly, SA treatment has been reported to enhance total phenols in fresh goji fruit, thereby helping to maintain antioxidant capacity and storage quality [80]. In addition, the SA application increased total phenolic content and alleviated postharvest quality deterioration in ‘France’ prune fruit through modulation of the antioxidant system [81].

Total protein, including various defense enzymes and other non‐enzymatic protein‐based compounds, plays a vital role in plant defense mechanisms [79]. Additionally, the role of proteins in plant‐induced resistance is well established [82]. This hypothesis is supported by previous findings showing that neonicotinoid insecticides can increase total protein content in plants [83]. Elevated levels of soluble proteins are closely associated with enhanced carbon dioxide (CO_2_) fixation, which in turn improves photosynthetic efficiency. Plants respond to herbivore attack by triggering coordinated defense strategies that involve the accumulation of defense‐related proteins [84]. This complex response begins with the perception of stress signals at the cellular level, followed by intracellular signal transduction, leading to the synthesis of defense molecules and their targeted transport to critical sites [85, 86]. Ultimately, the observed increases in photosynthetic activity and total protein content are governed by both the innate immunity of individual cells and systemic signaling processes initiated at infection sites [87].

Our findings regarding the enhancement of plant growth and fruit traits are consistent with previous reports [88, 89, 90]. Phosphite application has been shown to significantly improve vegetative growth, yield, and fruit quality in pepper [88]. In addition, salicylic acid (SA) increased both root fresh and dry weight, whereas potassium phosphite (PK) resulted in the highest fruit number, length, and diameter compared with other treatments [89]. Similarly, salicylic acid (SA) application enhanced leaf area and dry weight, while potassium phosphite (PK) exhibited comparable effects [90]. Phosphite plays a vital role in key physiological processes, including cell division, energy transfer, photosynthesis, enzyme regulation, sugar and starch metabolism, and nutrient transport [91]. Regarding fruit traits, potassium availability significantly influenced fruit number, size, and diameter by enhancing sugar translocation, carbohydrate synthesis, protein formation, and other physiological processes essential for plant growth and reproduction [92]. Imidacloprid (IMI) is a widely commercialized synthetic insecticide that has shown negative effects on many organisms [54]. As a neonicotinoid, IMI breaks down in plants to 6‐chloronicotinic acid, which is related to the SAR inducer isonicotinic acid [79]. Foliar applications of IMI have shown potential antioxidant properties, helping plants tolerate insect pest infestations [93]. Additionally, multiple IMI applications improved plant growth and yield, likely due to the establishment of systemic acquired resistance (SAR) [93, 94]. Imidacloprid (IMI) was reported to be less toxic to *Orius insidiosus* adults than bifenthrin [95] and more effective than several other insecticides against *Thrips tabaci* [96, 97]. Moreover, repeated applications of IMI enhanced plant growth even under low insect pressure, likely through improved physiological functioning and the induction of systemic acquired resistance (SAR) [79, 98, 99].

Phosphite enhances nutrient absorption, product quality, and tolerance to both biotic and abiotic stresses as a biostimulator [46]. In field and olfactometer assays, the responses of sucking pests to phosphite treatments were likely due to changes in volatile compounds released by pepper plants. Phosphite has been shown to induce rapid, transient changes in gene expression, triggering pepper defense mechanisms, including leaf lignification, cell wall thickening, and increased Beta synthesis [100]. Additionally, phosphite treatment increases 1,3‐glucanase and peroxidase enzyme activity, which can influence larval feeding performance [101]. While it is unclear whether phosphite deters ovipositing females or impacts larval survival, its effectiveness against *A. gossipi* may be linked to the activation of defense genes through both salicylic acid (SA) and jasmonic acid pathways [87, 101]. Similar to ‐aminobutyric acid, phosphite acts as a plant resistance inducer (PRI), stimulating defense enzyme, phytoalexin, and phenolic compound synthesis at low concentrations, while inhibiting pathogen growth at higher concentrations [102]. Phosphite has been reported to protect the photosynthetic apparatus by enhancing oxidative balance and improving physio‐biochemical attributes [103].

In the case of effective microorganisms (EMs), it is hypothesized that certain microbes can secrete a range of volatile and non‐volatile metabolites that either promote plant growth through various mechanisms or produce toxins that reduce the populations of *Aphis gossypii and Thrips tabaci*. Our findings are consistent with previous reports indicating that volatile organic compounds (VOCs) emitted by plant‐associated microorganisms represent an effective strategy for enhancing plant growth [104]. Notable VOCs such as 2,3‐butanediol, 3‐hydroxy‐2‐butanone (acetoin), 2‐pentylfuran, and dimethyl hexadecylamine have been shown to stimulate root and leaf development. Furthermore, additional volatile organic compounds (VOCs), including 2‐octanone, 3‐octanol, and 2,5‐dimethylfuran, have also been reported to contribute to plant growth promotion [105]. Beneficial microorganisms such as plant growth‐promoting fungi (PGPF), plant growth‐promoting rhizobacteria (PGPR), and arbuscular mycorrhizal fungi (AMF) enhance key plant traits, including shoot length, leaf area, nitrogen and chlorophyll content, and overall yield, while also improving plant tolerance to both biotic and abiotic stresses [106]. The reduction in *Aphis gossypii* and *Thrips tabaci* populations observed in this study may be attributed to toxic compounds produced during microbial colonization, a mechanism consistent with previous findings [107]. EMs are also known to induce the “rotation effect,” a phenomenon in which EMs regulate beneficial microbial communities while suppressing harmful ones, similar to the effects observed with crop rotation [108]. Previous studies have investigated the insecticidal activity of EMs against various agricultural pests, including *Pectinophora gossypiella* [45], *Spodoptera littoralis* [109], and *Earias insulana* [110], as well as their acaricidal effects on *Tetranychus urticae* [111] and *Bemisia tabaci* [46]. These studies consistently demonstrate the efficacy of EMs in pest control.

Regarding salicylic acid (SA), our results are consistent with previous findings demonstrating that SA enhances the activity of antioxidant enzymes [112]. In addition, SA activates plant defense–related genes, leading to the synthesis of antioxidant enzymes, steroidal glycoalkaloids, and the emission of volatile organic compounds that attract natural enemies of insect pests. These findings are consistent with previous studies [27, 53, 113, 114, 115, 116]. Salicylic acid (SA) has been shown to contribute to the activation of direct defense mechanisms against aphids [108]. Moreover, SA application has been reported to reduce insect pest populations across several cropping systems; for instance, SA treatment slightly reduced spotted bollworm populations in cotton [114], while foliar application under field conditions suppressed insect infestations more broadly [115]. In addition, combining SA with insecticides enhanced the control of whiteflies on cotton, allowing a reduction of pesticide use by up to 25%, an effect attributed to increased phenolic compound accumulation in leaves [116]. Exogenous application of SA has also been shown to suppress caterpillar feeding and delay the development of insect resistance [27]. Moreover, SA, a naturally occurring phenolic plant hormone, plays a crucial role in activating plant defense responses against pest attacks [53]. Recent studies have highlighted both lethal and sublethal effects of pest control agents on target insect species, underscoring the importance of assessing their impacts on beneficial insects, particularly natural predators that play a critical role in biological control [117, 118, 119]. Our hypothesis supports the notion that the tested agents exhibit favorable selectivity toward certain common predator species of *Aphis gossypii* and *Thrips tabaci*. This increased selectivity observed during the study period suggests a potential for enhancing Integrated Pest Management (IPM) programs by promoting the effective integration of biological control strategies [120, 121].

## 5 Conclusions

In conclusion, this study demonstrates that phosphite, effective microorganisms (EMs), and salicylic acid function as effective non‐conventional agents that can enhance plant defense responses and contribute to the suppression of key sucking pests in sweet pepper under integrated pest management (IPM) conditions. When applied prior to pest infestation and evaluated under threshold‐based dinotefuran intervention, these agents significantly reduced populations of *Aphis gossypii* and *Thrips tabaci* over two consecutive growing seasons.

Importantly, the tested agents exhibited favorable selectivity toward beneficial predators, indicating their compatibility with biological control components of IPM programs. Rather than replacing chemical insecticides, these agents act as complementary tools that may reduce reliance on repeated insecticide applications and support more sustainable pest management strategies. Their ability to activate plant defense mechanisms and maintain predator populations highlights their potential value in integrated, reduced‐risk pest control systems.

## Funding

The Open Access Publication Fund of Humboldt‐Universität zu Berlin funded the article processing charge. This work was supported by the Deanship of Scientific Research, Vice Presidency for Graduate Studies and Scientific Research, King Faisal University, Saudi Arabia (KFU260217).

## Conflicts of Interest

The authors declare no conflicts of interest.

## Supporting information




**Supporting File**: gch270093‐sup‐0001‐SupMate.docx.

## Data Availability

The data that support the findings of this study are available in the supplementary material of this article.
