# Supplementary material for: Non‐ Conventional Agents Enhance Sweet Pepper (Capsicum annuum L. var. annuum) Defense against Aphis Gossypii, Thrips Tabaci, and Their Predators Chrysoperla Carnea and Orius Insidiosus
Source: Glob Chall. 2026 Jan 31;10(2):e00590. doi: 10.1002/gch2.202500590 (PMC12860888; doi:10.1002/gch2.202500590)
Supplement: Supplementary file 1 — Supporting File: gch270093‐sup‐0001‐SupMate.docx. [file GCH2-10-e00590-s001.docx]

**Supporting Information for the manuscript entitled:**

Non- Conventional Agents Enhance Sweet Pepper (*Capsicum annuum* L. var. *annuum*) Defense Against *Aphis gossypii*, *Thrips tabaci*, and Their Predators *Chrysoperla carnea* and *Orius insidiosus*

**Main Correspondence:** Amged El-Harairy ([elharaam@hu-berlin.de](mailto:elharaam@hu-berlin.de))

**Co-Correspondence:** Hossam S. El-Beltagi ([helbeltagi@kfu.edu.sa](mailto:helbeltagi@kfu.edu.sa))

**Table (1):** Effect of microbial and chemical agents on some plant physiological and biochemical parameters of sweet pepper

| Treatment | Total Chlorophyll (mg/g FW) | Macro- elements  (mg/g FW) | |  | Total phenolics  (µg/ml) | Total protein  (mg/g) | |
| --- | --- | --- | --- | --- | --- | --- | --- |
|  |  |  | |  |  |  |  |
|  |  | N | P | K |  | Leaf | Fruit |
| IMI | 12.09 | 1.96 | 0.65 | 1.71 | 6307.05 | 3.66 | 4.12 |
|  | 12.1 | 1.95 | 0.66 | 1.7 | 6308 | 3.65 | 4.1 |
|  | 12.13 | 1.97 | 0.65 | 1.72 | 6309.5 | 3.66 | 4.12 |
| PK | 16.22 | 2.17 | 0.69 | 1.65 | 7199.01 | 4.32 | 5.33 |
|  | 16.18 | 2.17 | 0.69 | 1.66 | 7200.77 | 4.33 | 5.33 |
|  | 16.26 | 2.18 | 0.68 | 1.67 | 7201.16 | 4.34 | 5.31 |
| EMs | 13.11 | 2.01 | 0.52 | 2.02 | 6493.02 | 4.18 | 4.9 |
|  | 13 | 2.02 | 0.52 | 2.01 | 6490 | 4.17 | 4.92 |
|  | 13.2 | 2.03 | 0.53 | 2.02 | 6495.5 | 4.19 | 4.91 |
| SA | 16.82 | 2.63 | 0.78 | 2.06 | 7415.76 | 5.15 | 6.6 |
|  | 16.77 | 2.61 | 0.8 | 2.07 | 7510.88 | 5.16 | 6.6 |
|  | 16.9 | 2.64 | 0.79 | 2.05 | 7419.99 | 5.14 | 6.61 |
| Water-Sprayed Untreated Check (Control) | 12.88 | 1.82 | 0.52 | 1.59 | 6231.01 | 3.14 | 3.88 |
|  | 12.9 | 1.81 | 0.51 | 1.59 | 6231 | 3.15 | 3.87 |
|  | 12.91 | 1.8 | 0.52 | 1.58 | 6231.09 | 3.15 | 3.86 |

**Table (2):** Effect of microbial and chemical agents on some plant growth and fruit yield parameters of sweet pepper

| Treatment | Plant parameters | | | | | Fruit parameters | | | |
| --- | --- | --- | --- | --- | --- | --- | --- | --- | --- |
|  | Plant length (cm) | No. of leaves | No. of branches | Plant FW  (g) | Plant  DW  (g) | No. Fruits/ plant | Fruit Length  (cm) | Fruit Diameter  (cm) | |
| IMI | 43.11  43.10  43.09 | 59.00  58.00  57.00 | 2.52  2.50  2.55 | 433.18  430.30  430.00 | 206.08  206.46  205.44 | 52.17  51.11  52.38 | 6.98  6.95  6.99 | | 4.01  4.02  4.03 |
| PK | 46.22  46.20  46.18 | 78.00  78.00  77.00 | 4.11  4.10  4.09 | 604.22  605.90  609.55 | 291.10  290.00  289.10 | 56.32  56.22  56.82 | 8.06  8.06  8.05 | | 4.78  4.77  4.75 |
| EMs | 42.09  42.00  42.10 | 72.00  73.00  72.00 | 3.23  3.20  3.25 | 577.45  578.77  279.99 | 250.90  248.48  252.25 | 53.14  51.99  55.50 | 7.83  7.88  7.85 | | 4.55  4.52  4.50 |
| SA | 46.82  46.90  46.50 | 84.00  83.00  85.00 | 4.45  4.40  4.45 | 635.76  634.40  630.18 | 301.41  300.10  299.37 | 51.43  52.89  50.15 | 6.44  6.40  6.44 | | 3.87  3.80  3.84 |
| Water-Sprayed Untreated Check (Control) | 42.40  42.40  42.42 | 40.00  40.00  39.00 | 2.14  2.10  2.12 | 402.11  402.10  404.19 | 192.70  192.80  192.50 | 23.10  23.14  23.10 | 5.12  5.10  5.09 | | 3.66  3.65  3.64 |

**Table (3):** The effect of tested agents against *Aphis gossipi* during the 2022 and 2023 seasons*.*

| Treatment | Reduction % | | | | | |
| --- | --- | --- | --- | --- | --- | --- |
|  | 2022 | | | | 2023 | |
|  | 1st | 3rd | 7th | 1st | 3rd | 7th |
| IMI | 93.14  92.44  93.22 | 89.90  90.88  91.11 | 76.37  73.18  75.71 | 92.18  93.14  89.07 | 89.66  88.80  84.40 | 71.15  78.01  62.57 |
| PK | 95.55  93.66  94.80 | 92.54  89.37  90.59 | 80.68  74.62  80.58 | 95.88  94.18  92.99 | 92.19  90.23  91.25 | 76.05  75.09  79.40 |
| EMs | 94.44  88.50  89.19 | 92.40  85.17  85.86 | 64.14  67.62  66.30 | 84.17  87.70  85.76 | 81.29  84.40  81.02 | 77.87  74.41  64.64 |
| SA | 84.40  86.16  86.09 | 83.35  81.10  82.82 | 72.46  69.80  74.63 | 88.17  85.45  89.18 | 80.13  81.33  86.60 | 66.12  72.53  70.71 |
| Water-Sprayed Untreated Check (Control) | 90.18  91.17  89.17 | 85.20  84.17  80.09 | 63.30  62.86  58.77 | 83.67  87.18  77.19 | 81.90  80.15  73.37 | 60.93  63.37  59.41 |

**Table (4):** The effect of tested agents overlapping with dinotefuran against *Thrips tabaci* during the 2022 and 2023 seasons.

| Treatment | Reduction % | | | | | | |
| --- | --- | --- | --- | --- | --- | --- | --- |
|  | 2022 | | | | | 2023 | |
|  | 1st | 3rd | 7th | 1st | 3rd | | 7th |
| IMI | 96.60  95.50  97.58 | 93.30  92.27  93.17 | 77.01  88.32  86.15 | 97.90  98.80  97.69 | 95.50  96.69  94.85 | | 86.99  86.54  74.71 |
| PK | 6.43  96.79  96.15 | 90.08  91.44  90.26 | 73.38  70.64  86.92 | 95.70  96.49  95.50 | 90.18  91.79  94.66 | | 79.32  77.82  79.67 |
| EMs | 97.58  95.18  96.17 | 90.25  90.01  89.80 | 69.24  64.41  65.13 | 95.58  94.39  94.65 | 90.58  90.30  91.49 | | 60.02  56.81  59.89 |
| SA | 96.60  94.30  95.28 | 89.12  89.45  88.60 | 74.83  60.75  60.32 | 94.14  92.16  95.19 | 91.38  90.56  91.43 | | 64.47  59.71  69.13 |
| Water-Sprayed Untreated Check (Control) | 88.17  85.50  84.39 | 84.28  80.18  81.15 | 54.05  59.62  58.11 | 85.16  84.05  86.67 | 80.80  81.10  84.10 | | 53.46  57.33  72.50 |

**Table (5):** An indirect effect of tested agents overlapping with dinotefuran against the predator *Chrysoperla carnea* during the 2022 and 2023 seasons*.*

| Treatment | Reduction % | | | | | |
| --- | --- | --- | --- | --- | --- | --- |
|  | 2022 | | | | 2023 | |
|  | 1st | 3rd | 7th | 1st | 3rd | 7th |
| IMI | 30.15  31.56  29.90 | 26.92  28.29  27.44 | 15.32  16.80  14.33 | 31.17  32.77  31.50 | 20.54  24.14  19.78 | 16.45  17.88  17.72 |
| PK | 26.26  24.51  22.17 | 20.14  14.35  12.77 | 10.21  8.84  7.75 | 21.15  27.77  26.22 | 19.95  20.02  10.09 | 9.60  11.94  9.26 |
| EMs | 18.67  19.90  17.15 | 10.78  15.08  14.10 | 7.21  9.36  9.10 | 17.66  17.65  18.65 | 9.04  11.65  8.44 | 6.63  6.01  8.19 |
| SA | 14.22  14.03  15.05 | 9.99  11.37  10.66 | 5.46  4.90  4.39 | 14.14  13.23  14.83 | 10.04  8.56  11.07 | 3.12  2.69  3.92 |
| Water-Sprayed Untreated Check (Control) | 39.90  38.81  40.17 | 30.06  27.81  31.78 | 13.35  13.33  11.99 | 37.68  40.34  39.76 | 27.09  24.25  27.85 | 11.88  12.36  13.90 |

**Table (6):** The effect of tested agents overlapping with dinotefuran against the predator *Orius insidiosus*

| Treatment | Reduction % | | | | | | |
| --- | --- | --- | --- | --- | --- | --- | --- |
|  | 2022 | | | | | 2023 | |
|  | 1st | 3rd | 7th | 1st | 3rd | | 7th |
| IMI | 50.29  40.23  41.37 | 40.28  30.26  30.39 | 19.86  17.71  11.31 | 35.52  37.29  30.49 | 28.76  27.48  21.69 | | 9.37  9.57  8.81 |
| PK | 39.35  39.14  38.56 | 31.45  34.39  33.12 | 16.51  13.71  16.31 | 26.18  25.75  27.60 | 17.67  17.75  20.17 | | 10.31  8.67  16.91 |
| EMs | 34.29  35.35  34.45 | 30.50  28.75  27.18 | 9.91  11.50  10.73 | 32.20  31.73  30.08 | 27.45  28.31  25.35 | | 16.11  18.23  13.39 |
| SA | 22.50  20.37  21.27 | 10.48  9.88  6.34 | 9.42  8.18  11.63 | 21.56  22.38  18.89 | 14.90  16.38  11.37 | | 6.08  7.30  6.61 |
| Water-Sprayed Untreated Check (Control) | 51.87  52.76  53.28 | 43.39  45.87  40.27 | 25.94  27.37  29.18 | 41.25  37.57  41.80 | 32.18  30.90  36.48 | | 21.97  20.33  21.47 |
